# A novel classification framework for genome-wide association study of whole brain MRI images using deep learning

**DOI:** 10.1371/journal.pcbi.1012527

**Published:** 2024-10-15

**Authors:** Shaojun Yu, Junjie Wu, Yumeng Shao, Deqiang Qiu, Zhaohui S. Qin, for the Alzheimer’s Disease Neuroimaging Initiative

**Affiliations:** 1 Department of Computer Science, Emory University, Atlanta, Georgia, United States of America; 2 Department of Radiology and Imaging Sciences, School of Medicine, Emory University, Atlanta, Georgia, United States of America; 3 University of Chicago, Chicago, Illinois, United States of America; 4 Department of Biostatistics and Bioinformatics, Rollins School of Public Health, Emory University, Atlanta, Georgia, United States of America; National University of Singapore, SINGAPORE

## Abstract

Genome-wide association studies (GWASs) have been widely applied in the neuroimaging field to discover genetic variants associated with brain-related traits. So far, almost all GWASs conducted in neuroimaging genetics are performed on univariate quantitative features summarized from brain images. On the other hand, powerful deep learning technologies have dramatically improved our ability to classify images. In this study, we proposed and implemented a novel machine learning strategy for systematically identifying genetic variants that lead to detectable nuances on Magnetic Resonance Images (MRI). For a specific single nucleotide polymorphism (SNP), if MRI images labeled by genotypes of this SNP can be reliably distinguished using machine learning, we then hypothesized that this SNP is likely to be associated with brain anatomy or function which is manifested in MRI brain images. We applied this strategy to a catalog of MRI image and genotype data collected by the Alzheimer’s Disease Neuroimaging Initiative (ADNI) consortium. From the results, we identified novel variants that show strong association to brain phenotypes.

## Author summary

Genome-wide association study (GWAS) is a powerful method to identify associations between genetic variants and traits such as height, weight and disease status. When applying to Magnetic resonance imaging (MRI) data, traditional GWAS methods often rely on simplified summaries of brain imaging data, potentially missing subtle but significant global patterns. We proposed and implemented a different strategy: training a machine learning model to distinguish MR images based on genetic variants. If MRI images labeled by the mutation status of a variant can be reliably distinguished using machine learning, we then hypothesized that this variant is likely to be associated with brain anatomy or function which is manifested in MRI brain images. By applying this method to data collected from the Alzheimer’s Disease Neuroimaging Initiative (ADNI), we found new genetic variants highly likely to affect brain phenotypes. This innovative approach not only handles high-dimensional imaging data more effectively but also captures complex, non-linear relationships between genetic variants and various brain traits, offering a fresh perspective on neuroimaging genetics.

## Introduction

Over the past decade, genome-wide association studies (GWASs) have been successfully applied to many diseases and traits and identified a large number of genotype-phenotype associations [1,2]. One notable success of GWAS is in the field of neuroimaging genetics, which aims to characterize, discover, and evaluate the association between genetic variations and brain imaging measurements [3]. Neuroimaging studies of twins and their siblings have revealed that numerous key brain structures are strongly influenced by genetic factors [4–11]. Recent advances in acquiring high-dimensional brain imaging and genome-wide data have provided unprecedented new opportunities to study the effect of genetic variations on a wide range of brain phenotypes. Exciting findings have been made using GWAS that connects genetic variants with brain functions and anatomical structures [3,12–20].

Despite many novel findings, almost all neuroimaging GWAS studies conducted so far use pre-determined hand-crafted summary statistics, named imaging quantitative traits (iQTs) or imaging-derived phenotypes (IDPs) [1], drawn from individual voxels [2] or a pre-specified Regions-of-Interests (ROI) of the brain [3–5] for univariate analyses. Such a practice might overlook important imaging characteristics that exist in full frame images, such as texture and subtle variations in the signal intensity of the images. Although methods have been developed to conduct GWAS on multiple traits simultaneously such as multifactor dimensionality reduction (MDR)-based and independent component analysis (ICA) [21]-based methods [6–9], they are not designed to analyze high-dimensional structured phenotypes such as images. Furthermore, these methods only explore linear relationships, which is inadequate for analyzing complex phenotypes like Magnetic Resonance (MR) images. These limitations originate from the fact that GWAS as we know it is built under the hypothesis testing framework.

**Fig 1:**
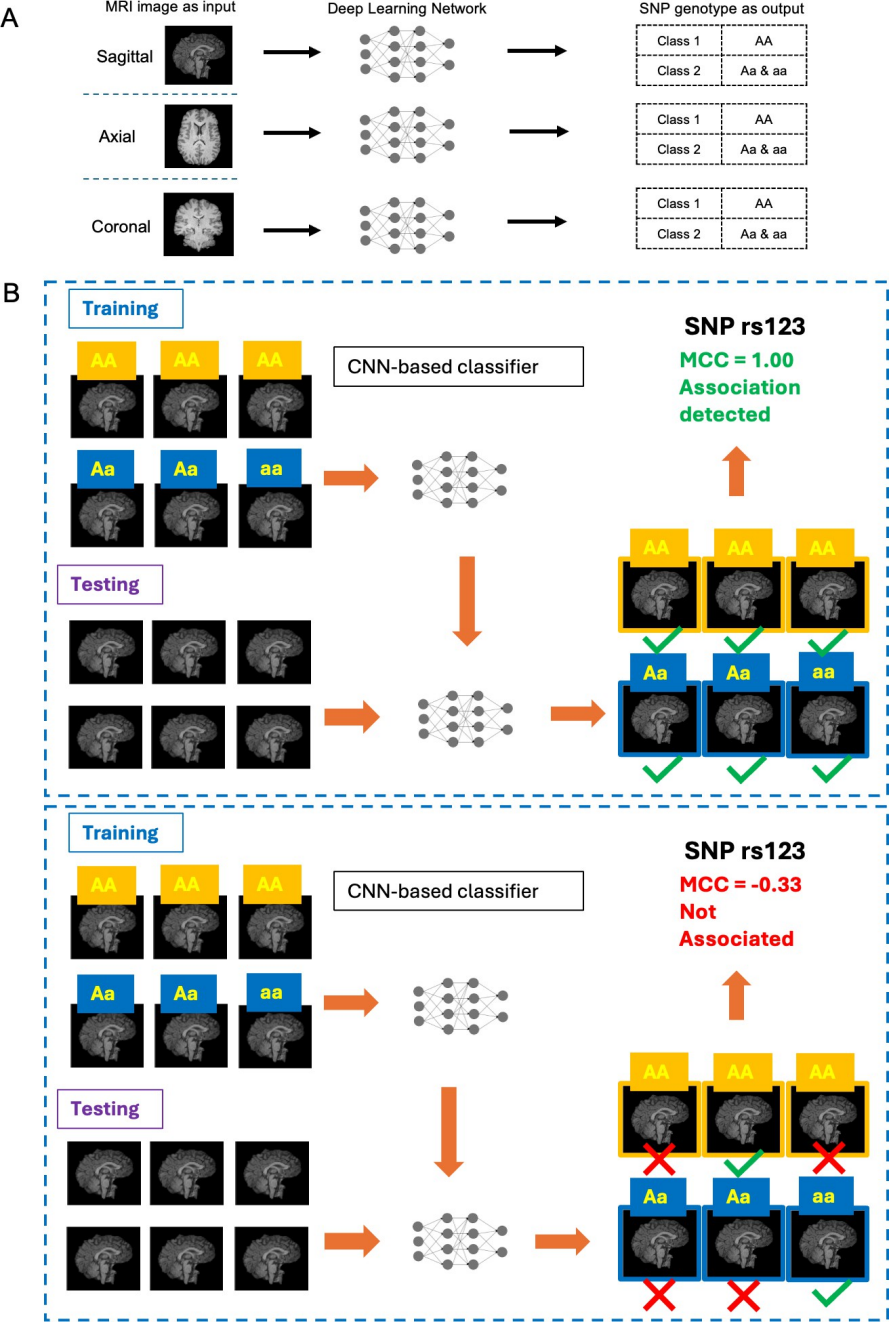
**Illustration of the proposed classification-based GWAS framework** (A) Schematic representation of the proposed classification-based GWAS framework for conducting association study on Magnetic Resonance (MR) images, where full-frame brain MR images from different directions—axial, coronal, and sagittal, serve as input and SNP genotypes treated as labels. Classification was carried out by convolutional neural network (CNN) models. (B) Detailed illustration of the classification-based framework for detecting the associations between brain MR images and SNPs. In the training stage, true labels are assumed known. Yellow label indicates class 1, blue label indicates class 2. In the testing stage, true labels are only used at the end to evaluate performance. Image with yellow frame indicates the image is predicted to be in class 1, image with blue frame indicates the image is predicted to be in class 2. Green check mark indicates a correct prediction, red “x mark” indicates an incorrect prediction.

In this study, we present an innovative alternative strategy: conducting GWAS under a classification framework. For each single nucleotide polymorphism (SNP), we use the genotypes of this SNP as the label to divide all images into distinct subgroups, and then train a convolutional neural network (CNN)-based classifier to separate different subgroups of images. The hypothesis behind our strategy is that if an SNP is associated with brain-related traits, then there is high likelihood that detectable differences exist between brain images belonging to different genotype groups of this SNP. And such differences, albeit subtle, can be detected by adequately trained machine learning algorithms. Please see Fig 1 for an illustration of our proposed strategy.

A fundamental advantage of our new strategy is to enable association study to be conducted on high-dimensional phenotypes in a conceptually simple and natural way. Compared to the classical hypothesis testing-based strategy, our method relies on a different set of criteria to identify genotype-phenotype association. Under the hypothesis testing framework, the goal is to test whether a specific model parameter is the same across the two populations. P-values were used to represent the level of difference. In contrast, under the supervised classification framework, the focus is placed on finding ways to better distinguish samples belonging to the two populations. A completely different set of performance measures was used.

Compared to existing GWASs, our proposed method boasts two key novelties. First, our method can handle ultra-high-dimensional traits. As an example, in this study, the dimension of the three phenotypes we studied is either 33,124 or 39,676. Second, our method can accommodate non-linear relationships between genotypes and phenotypes. Both pose strenuous challenges under the traditional hypothesis testing framework. But under the classification framework, deep learning-based classifiers have been routinely utilized in computer vision and demonstrated extraordinary performance. We believe this powerful deep-learning-based method is able to make novel discoveries under a GWAS context.

## Results

### 1. Simulation results

**Fig 2:**
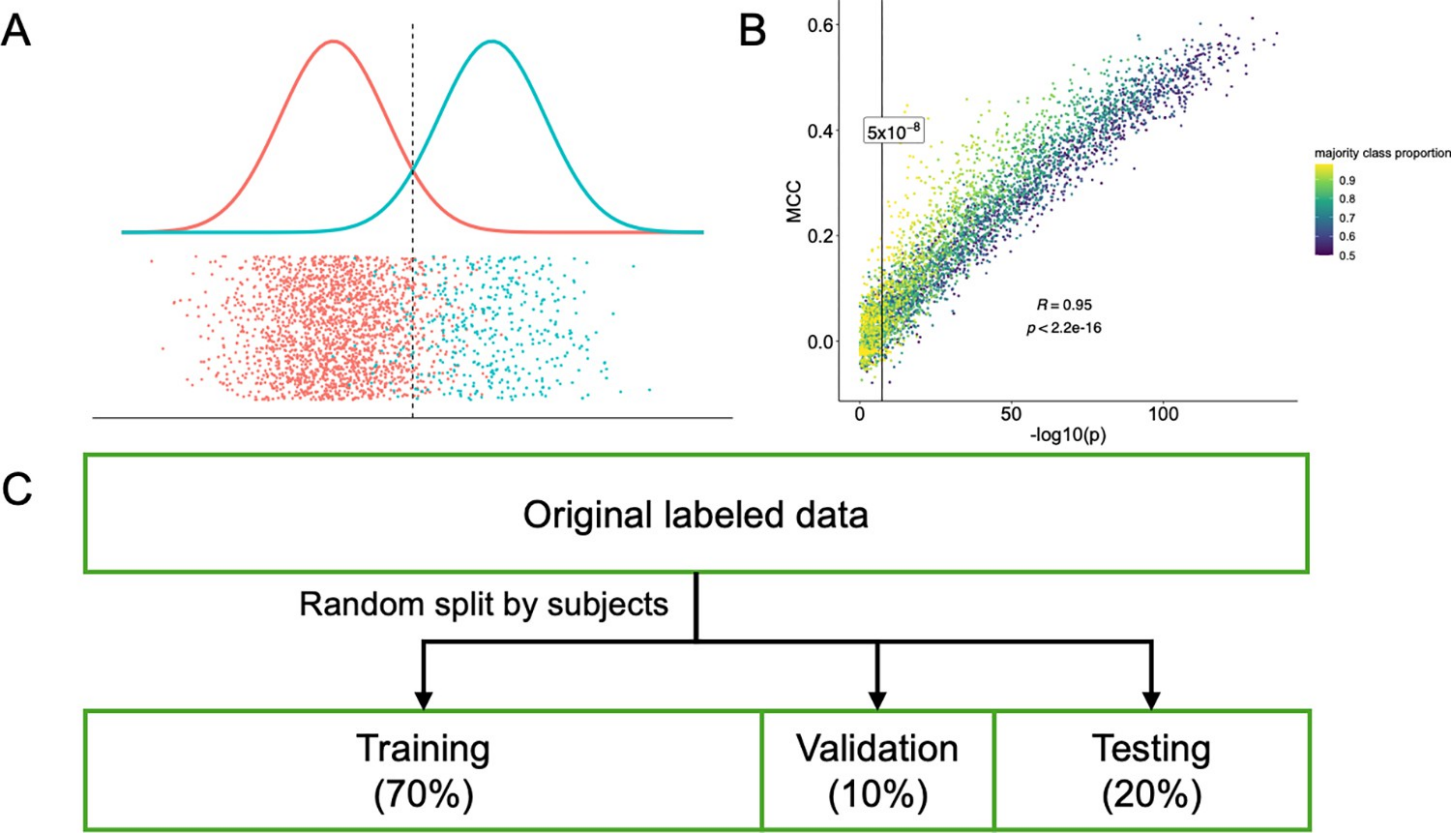
**Simulation study and data splitting.** (A) Schematic illustration of the simulation study, where classification and testing are performed on the same dataset. Data are generated from two distinct univariate normal distributions with the same variance but different means. (B) Comparing p-values of two-sided independent t-test with classification performance metrics Matthew’s Correlation Coefficient (MCC) in the simulation study. (C) Illustration of evaluation process of the classification task in which all images are randomly divided into training, validation and testing sets by subjects in the ratio of 7:1:2.

For the newly proposed strategy, it is of great interest and importance to understand how its result compares to the result obtained using the traditional hypothesis testing strategy, when applied to the same set of data. In order to understand the relationship between p-values and classification performance measures such as Matthew’s correlation coefficient (MCC) and macro F1 values, we conducted a series of simulation studies (Figs 2A and S2 and Table A in S1 Table). For easy comparison, simulations were conducted on univariate variables. To be specific, random variables were sampled from two distinct Normal distributions with varying parameters. From the results, we found that overall, there is good agreement in terms of correlation between the p-values (from the hypothesis testing framework, -log() transformed) and the MCC or macro F1 values (from the classification framework) with a strong correlation ranging from 0.94 to 0.96 (spearman correlation coefficient, Figs 2B and S2). We also notice that “moderate” classification measures (0.29≤*MCC*≤0.31) often correspond to highly significant testing results (p-values ranging from 10^−16^ to 10^−80^), especially when the sample size is large.

We also computed the Kullback-Leibler divergence (KLD) between the two Normal distributions in our simulation data. Not surprisingly, we found that across the board, there is a strong positive correlation between KLD and -log10(p) (S3 Fig, upper part) obtained from testing-based strategy, as well as a strong positive correlation between KLD and MCC (Matthew’s correlation coefficient) (S3 Fig, lower part) obtained from classification-based strategy. This is in line with the expectation that greater divergence between the two normal distributions corresponds to stronger association test result and easier classification task.

### 2. ADNI data

All the real data used in this study were obtained from the ADNI study (https://adni.loni.usc.edu/) [22]. The ADNI project recruited over 1,700 participants ranging from cognitively normal (CN) to patients with significant memory concerns (SMC), mild cognitive impairment (MCI), and Alzheimer’s disease (AD). ADNI aims to combine data from multiple imaging technologies, biomarkers, and clinical and neuropsychological assessment to study the progression of MCI and early AD. In this study, we primarily use the MRI and genotype data from ADNI 1 and ADNI 2. The detailed characteristics of the subjects are summarized in Table B in S1 Table. After quality control and preprocessing, we retain 13,722 2D MRI images and 313,267 SNPs from 1,009 subjects to be used in this study. Since ADNI is a longitudinal study, most participants contributed multiple MRI scans (S1A Fig). The demographic characteristics and the histogram of the data are presented in S1 Fig and Table B in S1 Table, respectively.

### 3. Overview of the method

We propose to conduct GWASs on full frame MR images of the brain under the classification framework. In classical GWAS, the phenotype, such as height, is treated as the response variable and genotypes are treated as explanatory variables. In our proposed method, genotypes are used to define classes (referred to as labels in the machine learning literature) and whole brain MR images are used as input. The underlying hypothesis posits that if a variant influences brain-related phenotypes, then different genotypes of the variant will exhibit distinct manifestations in the MR images. Such difference, albeit subtle, can be recognized by properly trained classifiers. In fact, different patterns in functional MRI have long been observed among patients with different genetic mutations. In a landmark study, Filippini et al. examined the effects of the APOE polymorphism on brain structure and function in 18 young healthy individuals who carried the APOE epsilon4 allele and 18 matched individual noncarriers [23]. They discovered that resting fMRI showed higher coactivation within the “default mode network” in epsilon4-carriers compared to noncarriers. This study provides compelling evidence that functional variants can influence patterns observed in fMRI data. In a more recent study, Roux et al. present strong evidence to support that multiple brain anomalies can be attributed to specific mutations. For example, leukodystrophy sparing periventricular white matter is linked to EARS2 mutations [24]. Under the regression context, our new strategy effectively reverses the status of response and explanatory variables. In this study, to mitigate computation burden, we used 2D MR images, chosen to be the middle slice images from three different planes: axial, coronal and sagittal, derived from the original 3D T1-weighted (T1w) MR images. The details of the data processing can be found in the Methods Section. Given the 2D MR images, we trained CNN-based classifiers aiming to distinguish MR images with different genotype labels. Here we adopted a binary classification scheme, one genotype class consisted of samples with the homozygous wildtype genotype denoted as AA; and the other category contains samples with the heterozygous genotype denoted as Aa and homozygous mutant genotypes denoted aa. All CNN models used in this study shared the same architecture and complexity such that their performances are comparable. In the end, we rank the SNPs based on their classification performance.

For each SNP, after removing subjects with missing genotype information at the SNP, the MR image collection was split into three subsets: a training set, a validation set, and a test set with the ratio of 7:1:2 (Fig 2C). The split is carried out in such a way that images from the same subject only appear in one of the three subsets to keep the three subsets independent. Then a CNN-based classification model was trained for a fixed number (30 in the present study) of epochs, and the best model during the training process was selected based on its performance measure on the validation set. During training, to save computing time, the process will be early stopped if there is no improvement in validation loss after ten epochs. Then, the final performance of the model was evaluated on the test set.

### 4. Model specification

Due to the fact that a massive number of classifiers need to be trained, we design our CNN model to be as simple as possible while maintaining decent performance. To help decide on the structure of the CNN model, we used the sex classification task as a benchmark. In addition to evaluating the models based on their classification performance, we also paid attention to the training time required to achieve this performance. We tested models with varying levels of complexity and compared with state-of-the-art CNN models including ResNet[25] and Xception[26]. Our results showed that modest CNN models produce excellent performance, with MCC 0.468, 0.387, and 0.619; macro F1 scores of 0.719, 0.682 and 0.805, in the axial, coronal, and sagittal planes, respectively, which is comparable to the state-of-the-art CNN models. Also, our modest CNN models required the least amount of time, at approximately 20 seconds to train for each task. In contrast, state-of-the-art CNN models such as ResNet and Xception require much longer training times, taking over 70 seconds to reach their best performance in training. These results are summarized in Table C in S1 Table. Overall, we found that modest CNN models can achieve comparable performance as the state-of-the-art CNN models while requiring significantly less training time, which makes them an attractive option for tasks where training time is a critical factor.

**Fig 3:**
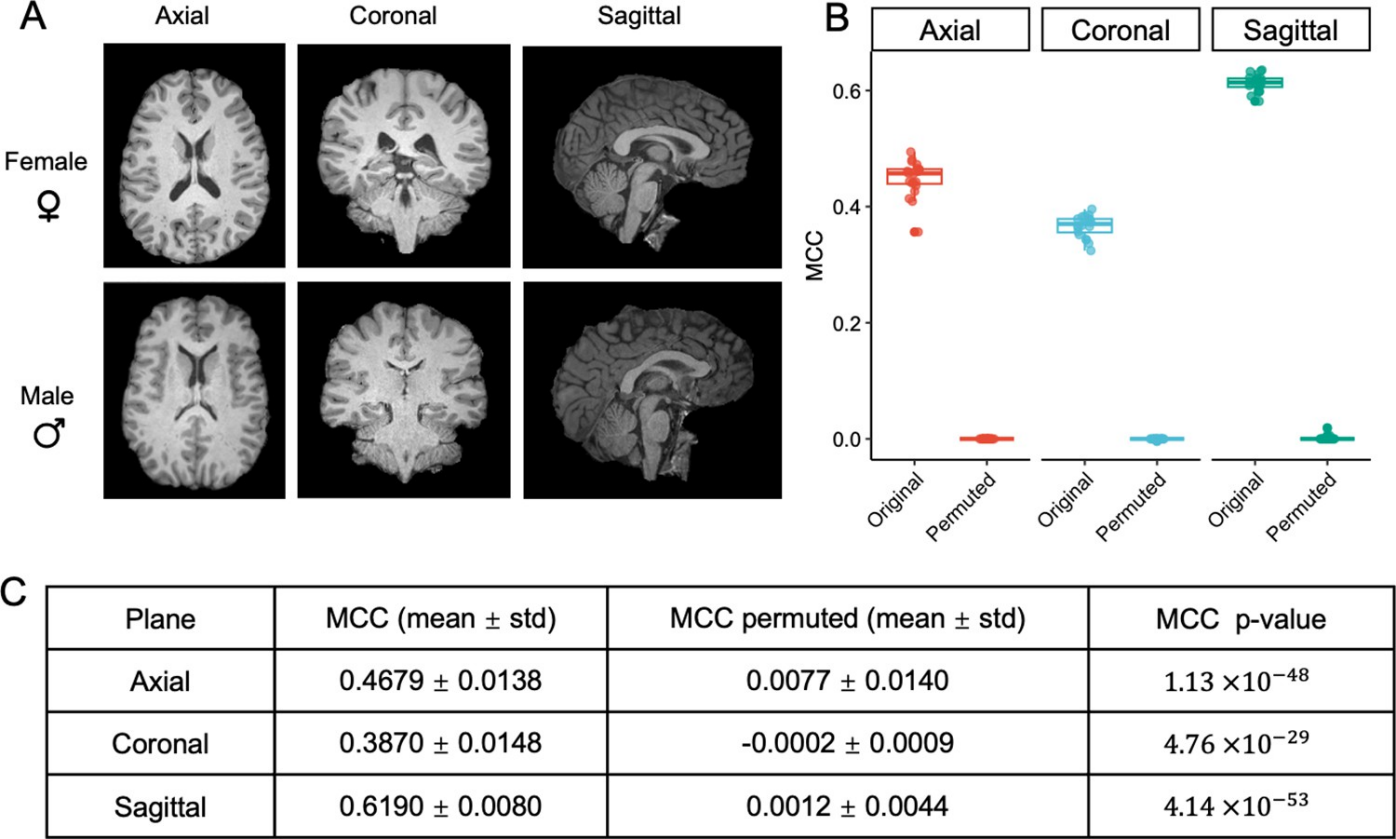
**Results from sex classification using 2D full-frame MR images of the brain** (A) Sample 2D full-frame brain MR images from three different planes—axial, coronal, and sagittal. (B) Side-by-side boxplots showing MCC values from sex classification in axial, coronal, and sagittal planes before and after randomly permuting the sex labels. (C) Summary statistics and p-values from the fine tuning step of sex classification using 2D full-frame MR images of the brain. The male and female icons used in this figure were obtained from a free online source (https://www.iconfinder.com/icons/7787571/female_woman_avatar_man_icon, https://www.iconfinder.com/icons/9034992/male_icon), and can be used under MIT license or Creative Commons (Attribution 3.0 Unported, https://creativecommons.org/licenses/by/3.0/).

For each classification task, in order to account for uncertainty in the performance of individual CNN runs and minimize potential biases caused by confounding factors, we adopted a fine-tuning strategy. Details of the method are described in the Methods section. Essentially, we repeated CNN runs 20 times, and as comparison, we also performed permutation test 20 times and measured MCC and macro F1 scores each time. We then conducted two-sample t-tests to compare the two sets of performance measures and obtained p-values. Using sex as an example (Fig 3A), we found that CNN models achieved significantly better performance in classifying sex-labeled MR images versus classifying label-permutated MR images with p-values 1.13×10^−48^, 4.76×10^−29^, 4.14×10^−53^, for the three planes respectively (Fig 3B and 3C). These findings suggest that our CNN model is able to effectively capture features that distinguish MR images taken from males and females. In summary, our results demonstrate that the modest CNN model we adopted is able to successfully classify MR images with biologically meaningful labels such as sex.

### 5. GWAS results

**Fig 4:**
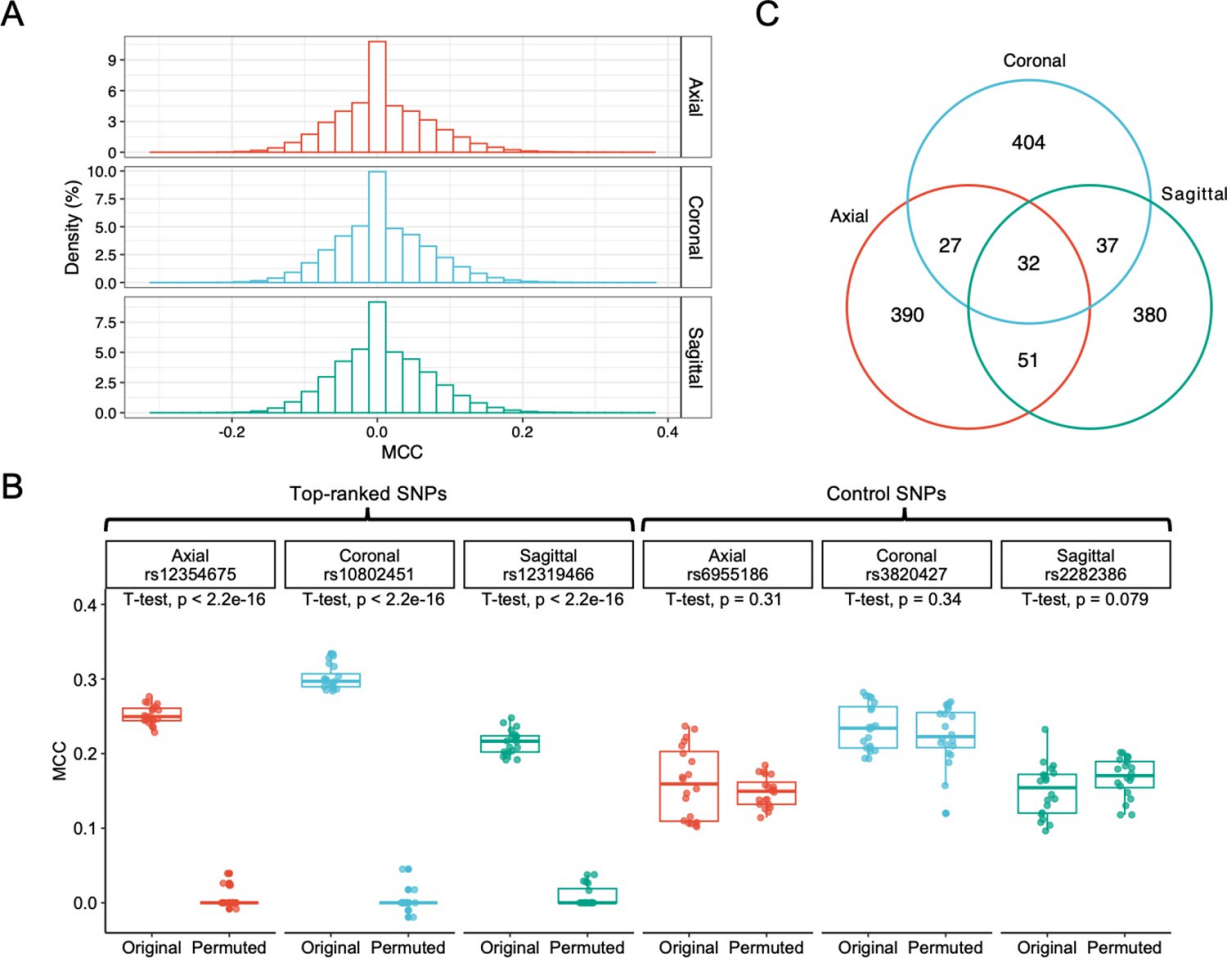
Main results from classification-based GWAS applied to Alzheimer’s Disease Neuroimaging Initiative (ADNI) MR image data. (A) Histograms of all MCC values derived from the GWASs conducted in each of the axial, coronal, and sagittal planes. (B) Illustration of the difference in the side-by-side boxplots between top-ranked SNPs and randomly selected low-ranked SNPs obtained from the GWAS conducted on three different planes—axial, coronal, and sagittal. The boxplot shows MCC values for classifying genotypes of the SNP, before and after randomly permuting the genotype labels. (C) Venn diagram displaying the number of overlapping SNPs among the three lists of top 500 SNPs ranked by MCC in the in the axial, coronal, and sagittal planes.

Using MRI image slices from three different planes—axial, coronal and sagittal, we trained three different models for each SNP and measured their classification performance on the test set to identify potential associations between SNPs and brain MR images. Two different criteria were used: MCC and macro-F1 score. The performance results for all SNPs are presented in Table D in S1 Table. A histogram was constructed to visualize the distribution of MCC scores for all SNPs in different planes (Fig 4A). The histogram showed that the distribution of MCC values was approximately normal with mean around 0, meaning that for most SNPs, genotype-labeled full-frame brain MRI images cannot be well classified using CNN-based classifiers, which is expected.

Using the MCC criterion, we selected the top 500 SNPs with the highest MCC values in each of the three different planes, which corresponds to top 0.018 percentile. From the Venn’s diagram (Fig 4C) we saw that most of the SNPs are unique (with only 32 SNPs that appear in all three lists), meaning that using 2D images derived from different planes leads to unique association findings. Among the three lists of top 500 SNPs, 1,321 are unique (referred to as the 1,321 top SNPs hereafter). To select the very top among them, we apply the fine-tuning strategy, and rank these SNPs according to the two-sample t-test p-values. Fig 4B shows examples comparing top-ranked and control SNPs in three different planes. Table 1 summarizes the top 20 SNPs ranked by the fine-tuning p-values.

**Table 1:** 20 Top SNPs significantly associated with full-frame MR image of the brain.

SNP	Plane	MCC	MCC P-value*	Context	Gene	Phenotypes
rs12354675	Axial	0.25	2.38×10^−40^	upstream gene variant	*NPFFR1*	Depression; **Right superior parietal**
rs10802451	Coronal	0.30	4.06×10^−39^	intron variant	*AHCTF1*	Depression; **Mean Pallidum**
rs998461	Axial	0.22	3.26×10^−37^	regulatory region variant	\	Insomnia
rs2253927	Coronal	0.24	7.88×10^−37^	intergenic variant	\	Alcohol dependency; **Cerebellar vermal lobules**
rs1198414	Axial	0.35	2.30×10^−36^	intergenic variant	\	Body Mass Index
rs6838213	Axial	0.33	9.73×10^−35^	intergenic variant	\	Neuroticism;
rs3812663	Axial	0.26	2.43×10^−34^	intergenic variant	\	Epilepsy; Alcohol dependency
rs17102716	Coronal	0.23	1.51×10^−33^	intron variant	*CTC-367F4*.*1*	**Right pericalcarine;** Insomnia
rs7768422	Axial	0.33	1.56×10^−33^	intron variant	*SLC22A16*	**Left isthmus cingulate;** Autism spectrum disorder
rs12319466	Sagittal	0.22	1.91×10^−33^	non-coding transcript variant	*RP11-588H23*.*3*	Cognitive; Schizophrenia
rs981684	Coronal	0.21	2.11×10^−33^	missense variant	*TMEM99*	**Right caudal middle frontal**
rs16903930	Axial	0.26	3.01×10^−33^	intron variant	*EGFLAM*	**Left pars triangularis**
rs2626192	Axial	0.30	3.29×10^−33^	regulatory region variant	*LINC01193*	\
rs6703902	Coronal	0.22	7.10×10^−33^	intergenic variant	\	Neuroticism; **Right rostral middle frontal**
rs4895172	Sagittal	0.24	7.21×10^−33^	intron variant	*CTC-546K23*.*1*	Worrier / anxious feelings; **Right accumbens area**
rs596985	Coronal	0.38	1.08×10^−32^	intron variant	*ROR1*	Depression; Cognitive
rs11845184	Axial	0.34	1.84×10^−32^	intron variant	*TSHR*	Neuroticism
rs11746806	Coronal	0.32	2.43×10^−32^	non-coding transcript variant	*RP11-1023L17*.*1*	Hair color
rs7982701	Coronal	0.25	3.60×10^−32^	intron variant	*ZMYM2*	Cognitive; **Mean Putamen**
rs4242182	Axial	0.29	1.12×10^−21^	intron variant	*MSX2*	Hair color; **Right superior frontal**

* For each SNP, we repeated CNN runs 20 times, and permutation test 20 times and measured MCC each time. We then conducted a two-sample t-test to compare the two sets of performance measures and obtained p-values

When using the macro F1 score, 1,301 of the top 500 SNPs identified across the three planes were unique. 957 SNPs appeared in the top 500 SNP lists ranked by both MCC and F1 (Tables E, F, and G in S1 Table). S4 Fig presents the top SNPs ranked by macro F1 and their annotations.

#### Known associations of the top SNPs

**Fig 5:**
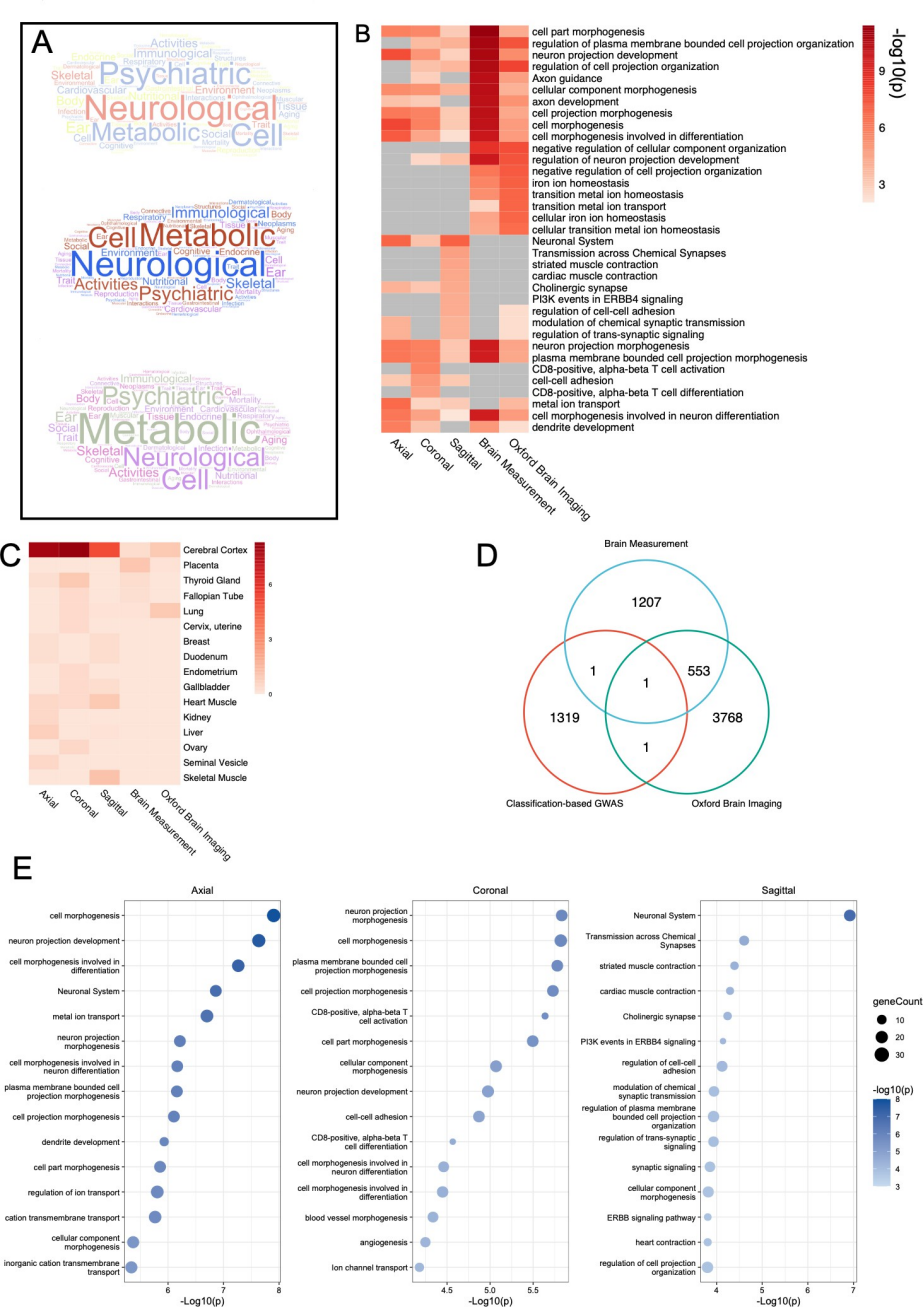
Exploring biological properties of genes close to the top SNPs. (A) A word cloud showing the major domains of phenotypes show up in PheWAS analyses of the 500 top-ranked SNPs identified from three different planes (from top to bottom): axial, coronal and sagittal. (B) The heatmap showing the enrichment levels (measured by -log transformed p-values) of GO terms and pathways from five lists of top-ranked genes. The first three lists correspond to top genes from our studies of classifying full-frame brain MR images from three different planes—axial, coronal, and sagittal. The fourth set was sourced from the GWAS catalog, the fifth one was acquired from the Oxford Brain Imaging Genetics (BIG) Server. Top 10 most enriched pathways identified from each of the five gene lists were used to generate this heatmap. A grey color indicates that the p-value is not significant (p > 0.05). (C) A heatmap comparing the enrichment (measured by -log transformed p-values) of the expression levels of the same five lists of top-ranked genes from a panel of 16 tissues using the TissueEnrich tool. (D) Venn diagram showing overlaps among top SNPs from three different sources: proposed classification-based GWAS, Brain Measurement (GWAS Catalog, 1762 SNPs) and the Oxford BIG (4323 SNPs). (E) Gene Ontology (GO) term and pathway enrichment analyses results from the GWASs conducted in each of the axial, coronal, and sagittal planes.

To understand the properties of the top SNPs identified, it is of great interest to learn what traits they are associated with according to existing GWASs. To find out, we queried the top SNPs using the GWASATLAS [27] tool and the detailed annotation results are presented in Tables H, I and J in S1 Table. Among the 1,321 top SNPs, we found that 859 (65.0%) of them were associated with one or more known phenotypes at P < 0.01. Among the known phenotypes that these SNPs are linked to, many are neurological, psychiatric, or developmental-related such as depression, neuroticism, epilepsy, autism spectrum disorder and schizophrenia (Fig 5A). It is particularly noteworthy that 11 out of the top 20 SNPs (55%) were already identified in previous studies as being associated with certain brain iQTs (bold text in Table 1) such as right superior parietal and mean pallidum.

Further exploration into the genetic functions revealed that genes proximal to most of these 20 SNPs, have been previously highlighted in research for their substantial roles in brain and neurons. For example, the SNP with the most significant p-value is upstream of *NPFFR1*, also known as *GPR147*, which is a G protein-coupled Neuropeptide FF Receptor belonging to the RFamide receptor family. NPFFR1 plays a pivotal role in modulating stress and anxiety responses. In a recent study, Shen et al. found that the expression of the *Npffr1* gene is up-regulated in mice exposed to chronic unpredictable mild stress (CUMS) that exhibited depression-related behaviors [28].

In another example, RP11-588H23.3, is a long non-coding RNA where the 10^th^ most significant SNP is located within. In a recent study, Gudenas and Wang found that the expression of RP11-588H23.3 is highly correlated with *CA2*, a key gene associated with intellectual disability [29].

Another SNP on the list, rs11845184, is located in the intron of the *TSHR* gene, which encodes the receptor for the thyroid-stimulating hormone (TSH). TSHR plays an important role in the formation of neuroticism and aggression development [30]. It is also implicated in the actions of thyroid hormones, which are indispensable for normal brain development. These hormones facilitate neurogenesis, synaptogenesis, and myelination, as well as the differentiation and migration of neuronal and glial cells [31]. GWAS Catalog [32] showed that this gene is associated with Tourette syndrome [33] and Schizophrenia [34].

Other SNPs also show interesting characteristics. For example, SNP rs16903930 is located in the intron of a gene named *EGFLAM* (EGF like, fibronectin type III and laminin G domains). GWAS Catalog showed that this gene is associated with multiple traits of interest including height [35], mathematical ability [36] and education attainment [37]. SNP rs596985 is located in the intron of a gene named *ROR1* (receptor tyrosine kinase like orphan receptor 1). GWAS Catalog showed that this gene is associated with multiple brain measurements [38], cortical surface area [39] and cortical thickness [39]. SNP rs7982701 is located upstream of a gene named *ZMYM2* (zinc finger MYM-type containing 2) which is shown to be associated with morphology-related traits such as body mass index [40] and adult body size[41] according to GWAS Catalog. SNP rs4242182 is located in the intron of *MSX2* (msh homeobox 2). GWAS Catalog showed that this gene is associated with cortical surface area [39], posterior cortical atrophy and Alzheimer’s disease[42], vertex-wise sulcal depth and brain morphology [39].

#### Pathway enrichment analysis

To understand which pathways the 1,321 top SNPs significantly affect, we located genes overlay or next to these SNPs and then conducted gene-based enrichment analyses using pathway enrichment tool Metascape [43], we found that several brain- and development-related functional categories are significantly overrepresented among these genes. These categories include the neuron system, neurogenesis, and cellular processes such as neuronal cell proliferation, differentiation, and maturation as well as cell adhesion (Fig 5B and 5E). These pathways are known to play critical roles in cellular growth, and differentiation in brain development.

#### Tissue enrichment analysis

To explore in which tissue the top genes are highly expressed, we conducted tissue enrichment analysis using TissueEnrich [44]. Remarkably, our results revealed that collectively, the 1,321 top genes express the highest in cerebral cortex, the tissue most related to brain in the 16-tissue panel (Fig 5C). Analysis using Enrichr [45] showed that brain tissues from individual donors are the most enriched among all tissue in the GTEx collection (S5 Fig). The enrichment results again confirm remarkably intimate relationships between top SNPs and brain functions and activities.

#### Compared to traditional GWAS on iQTs

For our new method, it is of interest to compare our results with those obtained using traditional neuroimaging GWASs, so that we can put our findings in context. To do this, we performed enrichment analyses on two sets of SNPs that were obtained from published neuroimaging GWAS conducted on iQTs. The first set was sourced from the GWAS catalog, the second was acquired from the Oxford Brain Imaging Genetics Server [20]. From each data source, we adopted a similar strategy by selecting the top 500 SNPs with significant associations to brain MR imaging measurements and mapping them to the nearest genes.

Among the five sets of SNPs obtained using various methods, we found that while the GWAS catalog and Oxford brain imaging SNP lists overlap substantially, there is little overlap between each of the three SNP lists (from three different planes) obtained by our method and the two aforementioned SNP sets discovered by conventional neuroimaging GWASs. This indicates that our method is able to discover novel brain-SNP associations that may be missed by conventional GWAS (Fig 5D). For pathway enrichment analyses, our results showed that all five sets of top genes showed significant and comparable enrichment in brain and neuron-related pathways. However, the level of enrichment in terms of tissue-specific expression showed that the three sets of top genes identified by our method are much higher in the cerebral cortex, the tissue most related to brain in the tissue panel, than the other two sets (Fig 5B). These findings provide further support for the conclusion that the SNPs identified by our novel GWAS strategy are highly relevant to the brain and likely to be involved in brain-related traits or diseases.

### 6. Saliency maps

**Fig 6:**
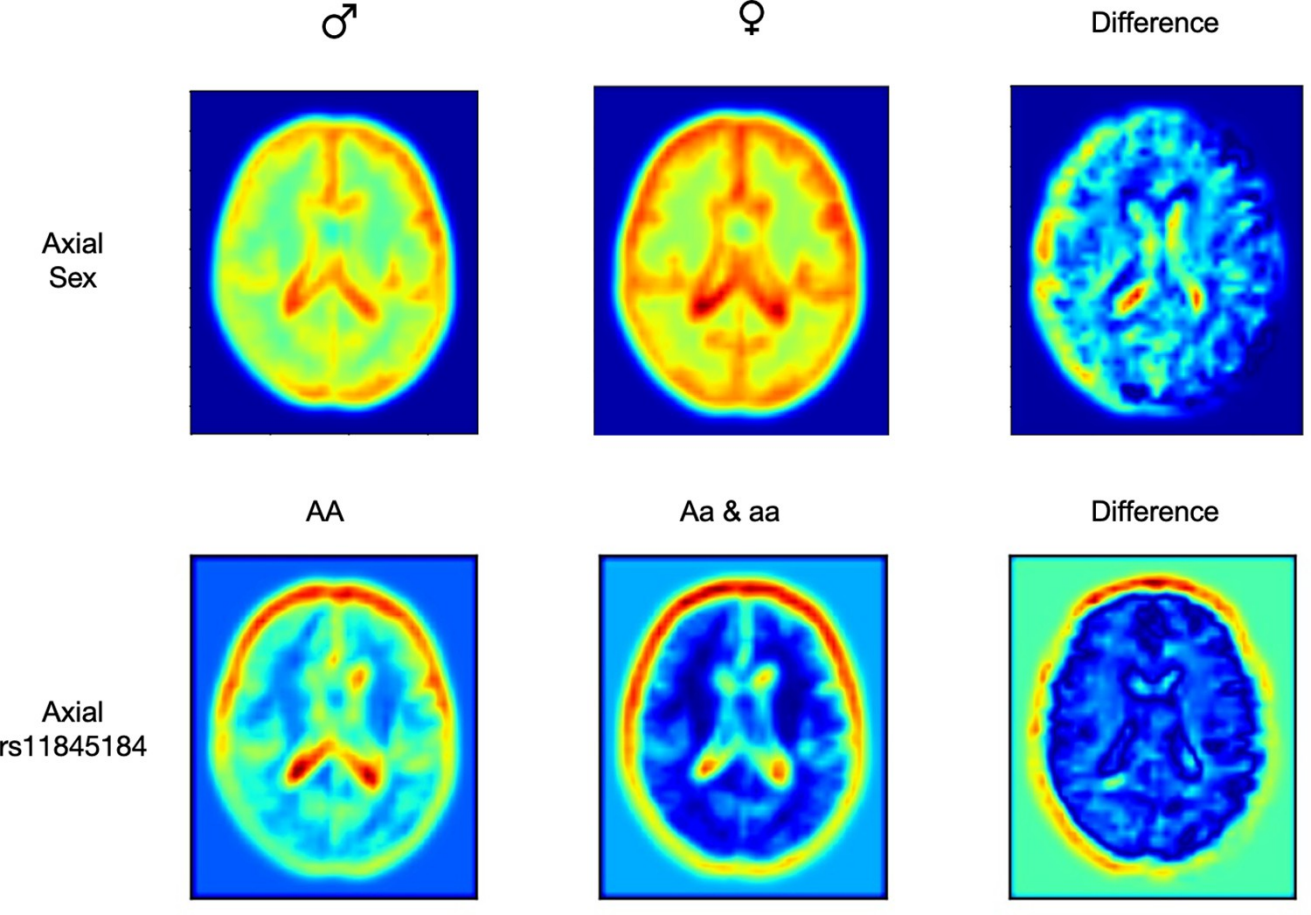
Saliency maps for the sex classification task and for the rs11845184 genotype classification task in the axial plane. The leftmost column shows the saliency map for male individuals, the middle column for female individuals, and the rightmost column for the difference between the two. The saliency maps in the first column depict the regions that are most important for the model to identify a male individual, while the second column illustrates the regions that are most important for the model to identify a female individual. The third column highlights the regions that are the most different between the two sexes, providing insight into the specific brain regions that are most differentiable.

In computer vision literature, saliency maps have been recognized as a powerful tool of visualizing attention, showing which parts of the input image contribute the most to the model’s prediction in CNN models [46]. Here we plan to use saliency maps to help us understand which parts of the brain the SNP is associated with. This is because highlighted regions in the saliency maps indicate brain regions that are highly relevant to the classification task. We used GradCAM++ [47] to visualize the attention over input images. A visual explanation for the corresponding class label is generated by combining the positive partial derivatives of feature maps of the last convolutional layer with respect to a specific class label. Model weights saved during training were loaded again to generate saliency maps for each of the two classes. Here we present the saliency maps for sex and the genotype of the SNP rs11845184 (Fig 6). Highlighted areas in the first two saliency maps indicate the brain regions that are closely related to the class label (sex or SNP genotypes). The third column shows the absolute difference between the saliency maps of the two labels, thereby allowing us to identify the areas that are most discriminative between the two classes. Such saliency maps can be applied to other SNPs to visualize the brain regions that are most relevant to genotypes of the SNP.

## Methods

### 1. Performance evaluation measures

Our novel strategy uses well established classification performance measures to represent the association between full-frame MR images and SNP genotypes. Two metrics were selected to evaluate the performance: MCC and macro F1 score. These two metrics are known to provide robust measure of the overall classification performance, even with imbalanced datasets. This is important because for many SNPs, genotypes contain the alternative allele has a low proportion[48]. The range of MCC is between −1 and +1. A coefficient of +1 represents a perfect prediction, random guesses produce MCC around 0. Let *tp*,*tn*,*fp* and *fn* represent the number of true positives, true negatives, false positives and false negatives, respectively. The MCC is defined as:

$$MCC\;=\;\frac{tp\;\times \;tn\;-\;fp\;\times \;fn}{\sqrt{\left(tp+fp)(tp+fn)(fn+fp)(tn+fn\right)}}$$ (1)

The F1 score is the harmonic mean of the precision and recall, defined as:

$$F1=2\times \frac{precison\times recall}{precison+recall}$$ (2)

The range of the F1 score is between 0 and 1, where 1 indicates perfect classification and 0 indicates either precision or recall is 0. The relative contribution of precision and recall to the F1 score is the same.

For our classification tasks, there is no clear distinction between the positive label and the negative label, and such labels are required to calculate the precision and recall. Therefore, we use the macro F1 score instead. The macro F1 score calculates an F1 score for either label designation and then uses their unweighted mean as the final result.

### 2. Simulation study

In this study, we propose a novel classification-based strategy to conduct GWAS on high-dimensional phenotypes. Compared to traditional GWAS, we use classification metrics such as MCC and macro F1 score to measure the strength of association, which is different from the metrics used in traditional GWAS, such as association test p-values, therefore it is important to understand how the two sets of performance measures compared to each other. To achieve this, we conducted simulation studies in which we applied both classification and hypothesis testing on the same dataset and compared their performance measures. To be specific, for each simulated dataset, univariate data points were randomly drawn from two Normal distributions: *N* (0, 1) and *N*(*μ*,1), where *μ* is fixed with values satisfy 0.2≤*μ*≤2, representing two different classes. For hypothesis testing, we used p-values from two-sample t-test to assess statistical significance. For classification, we used decision tree from R package rpart and compute the MCC and macro F1 score as performance measures. The simulation process is performed under different major class proportions (ranging from 0.5 to 0.98) and different values of *μ*. Since the size of *N* would affect the p-value, we also conducted this simulation study under different values of *N*, ranging from 3000 to 7000.

### 3. Genotyping data and processing

Genotyping for 1,009 participants was performed by investigators of the ADNI consortium using the Illumina Human 610-Quad, HumanOmni Express and HumanOmni 2.5 M BeadChips. PLINK was used to perform the standard quality control procedures for GWAS data. Exclusion criteria include SNP call rate < 95%, Hardy-Weinberg equilibrium test p-value < 1 × 10^−6^, minor allele frequency (MAF) < 5%. Additionally, only SNPs located on the autosomes are included in the present study.

### 4. Neuroimaging data and processing

Original MR image data used in the study are 3D T1-weighted images from ADNI 1 and 2. To prevent the model from inadvertently learning from non-brain regions, all MR images were skull-stripped using the bet tool from the FSL software package[49] (Oxford University, Oxford, UK). To account for variabilities in subject positioning and prescription of field-of-view, all high-resolution 3D T1-weighted images are normalized to the Montreal Neurological Institute (MNI) space[50]. To reduce the requirement for GPU memory and computational power, the images are resampled to 218 × 182 × 182 dimensions. To save computing time, we extract the middle slice from three different anatomical directions: sagittal, coronal, and axial using med2image (v.2.1.10, https://github.com/FNNDSC/med2image). The extracted images are in grayscale and standardized to be within 0 and 1.

### 5. Description of the classification-based GWAS

Our classification-based GWAS can be described as follows:

Let ***X***_***i***_ represents the MR image data for the *i*th subject, and *G*_*ij*_ represents the subject’s genotype for the *j*th SNP. Here we use *AA*,*Aa* and *aa* to represent homozygous wild type, heterozygous and homozygous mutant genotype, respectively. The following two formulas describes the key assumption we made in the method we propose:

$${X_{i}\sim E}_{j}\;if\;G_{ij}=AA$$ (3)

$$X_{i}\sim F_{j}\;if\;G_{ij}=Aa\;or\;aa$$ (4)

which means mathematically, MR image data follows two different distributions depending on the SNP genotype. Here *E*_*j*_ and *F*_*j*_ are high-dimensional distributions. We hypothesized that if SNP *j* is not associated with brain function or structure, then *E*_*j*_ = *F*_*j*_, otherwise *E*_*j*_≠*F*_*j*_. These two scenarios can be decided using binary classification.

### 6. CNN model architecture and model training

In our research, we processed each preprocessed slice from three different directions independently through a neural network. Our choice of network was LeNet [51], a simple yet effective Convolutional Neural Network (CNN) model structure, which was slightly modified for our needs. The modified LeNet50 model begins with a Conv2D layer, equipped with eight filters and a kernel size of 3x3. We used the ReLU activation function for this layer. This is followed by a MaxPooling2D layer with a pool size of 2x2, which reduces the spatial dimensions of the output volume. This pattern is reiterated twice, each time with Conv2D layers possessing 16 and 32 filters respectively and paired with additional MaxPooling2D layers. This design allows the model to learn more complex features from the input data. Following the final MaxPooling2D layer, the output is flattened into a one-dimensional array using a Flatten layer. This step is necessary to convert the 2D feature maps into a format suitable for input into the Dense layer that follows. Subsequently, a Dense layer with 128 units is applied, utilizing the ReLU activation function to add non-linearity to the network. To mitigate the risk of overfitting, a Dropout layer with a rate of 0.5 is introduced. This randomly sets half of the input units to zero at each update during training time, which helps prevent overfitting. The final layer is a Dense layer with two units, using a softmax activation function for binary classification. This function outputs a vector representing the probability distributions of a list of potential outcomes.

LeNet is one of the earliest CNN models, and despite its simplicity, it has demonstrated excellent performance with low computational cost. Given our need to train over 900,000 models, it was crucial to design the model as simply as possible while maintaining respectable performance.

For each SNP, we trained three individual models using images from three different directions. We divided the dataset for each binary classification task into three subsets: training, validation, and test sets, with a ratio of 7:1:2.

All models were trained on a server equipped with two TITAN RTX GPU cards. The average training time for a CNN model was approximately 20 seconds. Each model was trained for a maximum of 30 epochs at a learning rate of 1e-4. We selected the best model during training based on its performance on the validation set. The weights of the best model were saved and used to evaluate performance on the test set. Our models were developed using TensorFlow framework v2.4.3. Pre-trained weights on ImageNet were provided by the Keras framework v2.4.0. We used the pre-trained weights in our benchmark task–sex classification.

### 7. Permutation test of the classification performance

Performance measures such as MCC and macro F1 score are designed for a single classification task. But under the GWAS setting, the goal is to find out which ones are *classifiable* among hundreds of thousands of tasks, meaning that substantial and recognizable difference exists between the two genotype-labeled classes. Given that deep learning models are complex and dynamic, their performance may vary when the models are retrained. To mitigate the uncertainty, we developed a fine-tuning strategy.

The procedure has three steps. First, for each SNP, using the original training, validation and test set, we retrain the CNN model 20 times, providing a distribution of classification performance results. Next, we randomly shuffle the labels of the images, and train a CNN model with the same architecture on the shuffled data. This process is repeated 20 times, providing a null distribution of the classification performance results. The third step is to test whether the classification performance on the original data is better than that of the shuffled data by comparing the two sets of performance measures using a one-sided two-sample t-test. This procedure is performed for both MCC and macro F1 score. Due to heavy computing cost, we only applied the fine-tuning strategy to a small fraction of top-ranked SNPs.

### 8. Pathway, tissue enrichment for genes

We biological pathways enrichment analysis on Gene Ontology[52] biological process, Reactome Gene Sets[53] and KEGG Pathway[54] using Metascape[43](version 3.5, http://metascape.org), and the results were visualized with the ggplot2 R package. Gene expression tissue enrichment was performed using TissueEnrich[44] with human protein atlas data and Enrichr[45] with GTEx project data (https://gtexportal.org/home/). TissueEnrich and Enrichr are web-based tools that utilize a hypergeometric test to calculate the enrichment of a series of curated and pre-defined gene sets within a list of input genes.

### 9. Querying related databases

We used a series of databases to annotate the top SNPs and their putative target genes. For the context and gene information, we used the Ensembl Variant Effect Predictor[55]. The gene expression data were taken from the GTEx Project. In order to identify previous association studies for each independent locus, the genomic region containing these SNPs was first mapped to the hg38 version of the human genome using LiftOver, followed by a query against the GWAS Catalog database (https://www.ebi.ac.uk/gwas/) and GWAS atlas (https://atlas.ctglab.nl/) [27], choosing the nearest gene within 10kb of the SNP either upstream or downstream.

To put our results in context, it is of great interest to compare them to findings obtained from traditional iQT-based GWASs. To do this, we searched two leading databases for such findings: the GWAS Catalog and the Oxford Brain Imaging Genetics (BIG) Server (https://open.win.ox.ac.uk/ukbiobank/big40/) [20]. In GWAS Catalog, we use “brain measurement” (EFO ID: EFO_0004464) to query. A total of 1,742 SNPs were found to be associated with various brain iQTs (p-value threshold 1×10^−5^). For the BIG server, we included all the 4,323 associated (using the default p-value threshold 1×10^−7.5^) SNPs.

### 10. Saliency map construction

We visualized CNN model attention using the *tf_keras_vis* package (v0.8.1)[56]. This package provides various options for visualization, and we chose the Grad-CAM++[47] method to visualize the regions of the input image that contribute the most to the output value. Grad-CAM++ uses a weighted combination of the positive partial derivatives of the last convolutional layer feature maps with respect to a specific class score as weights to generate a visual explanation for the corresponding class label. During model training stage, we saved the best model weights and parameters. To construct the saliency maps, we first loaded the weights of the best model, we then replaced the SoftMax activation function in the last layer with a linear activation function. The score function for the saliency map was defined as the class labels and the test dataset was used to construct the saliency map for each SNP. Since the saliency map varies sample by sample, we take the numerical mean of all saliency maps derived from all samples in one class to get the class-level saliency map. Finally, the saliency maps were plotted using the Python matplotlib library with the “jet” color map.

## Discussion

In this study, we present a novel strategy that enables GWAS to be conducted on high-dimensional structured data such as brain MR images. The idea is to cast the GWAS on full-frame MR images under a classification framework in which the genotypes of an SNP define the classes (referred as labels in the machine learning literature), the full-frame MR images are treated as input. Under the new framework, determining the association between a SNP and brain-related traits relies on whether a reasonably trained classifier is capable of differentiating MR images belonging to separate genotype-defined classes. Compared to traditional GWAS which is hypothesis testing-based, our new classification-based strategy has two fundamental advantages: first, it can handle ultra-high dimensional phenotypes such as images and second, it is capable of picking up complex, nonlinear relationships between genotypes and phenotypes.

Given the same input data, the two strategies are designed to answer slightly different questions. Under the hypothesis testing framework, input data are assumed to be generated by two underlying distributions (often parametric), the goal is to examine the null hypothesis which states that there is no difference between the two distributions. In contrast, under the classification framework, the two groups are assumed to be distinct, the aim is to construct an effective classifier to distinguish two groups of data. The former, upon which the traditional GWAS is based, is a natural choice for univariate phenotypes, whereas the latter, handles multivariate traits much more elegantly. Using simulation, we showed that when applied to the same univariate trait, the two strategies return consistent results. Therefore, we believe the novel classification-based strategy can extend the powerful GWAS approach to multivariate phenotypes, especially, structured, ultra-high-dimensional phenotypes such as images.

In this study, we used sex classification as a benchmark to demonstrate the feasibility of our proposed method. We did this for several reasons. First, biological differences: It has been well-documented that males and females exhibit different brain structures, which can be observed through MRI imaging [57]. Studies have also shown that brain MRI can effectively classify sex based on these structural differences [58]. This biological basis makes sex classification a relevant and meaningful benchmark. Second, genotype analogy: genotypes of many SNPs in the sex chromosomes show drastically different frequencies in males and females, hence sex can serve as a surrogate of the genotypes of these SNPs, providing a classification task analogous to genotype classifications. Third, gold standard: the known sex of each subject provides a positive control, which allows us to rigorously assess and validate the performance of our classification model. This ensures that our model selection process is robust and reliable. By using sex classification as a benchmark, we aimed to validate our model’s ability to distinguish between biologically relevant categories using brain MRI data. This, in turn, helps to demonstrate the model’s potential effectiveness in genotype classification tasks.

A key advantage of taking the entire image of the brain as opposed to summary statistics as the phenotype is to allow the algorithm to discover important new brain traits in an unsupervised fashion, and capture global, higher-order nonlinear associations between variants and brain phenotypes. This could uncover genetic variant-brain associations that are difficult to be detected using traditional GWAS conducted on curated iQTs. To demonstrate its utility, we performed a large classification-based GWAS on brain MR images using publicly available datasets from the ADNI study [22] including 1,009 individuals. Among the top 20 most significant SNPs, 9 of them were not known to be related to any brain ROI. Through gene-set analysis, tissue and pathway enrichment analysis, we have demonstrated that many of the top SNPs have close relationships with brain development and the neuronal system. The saliency map offers clues about the relevant locations of the brain with which SNP genotypes are associated. Our results showed that our strategy has the potential to discover novel brain-related SNPs that have not been detected in previous studies.

Traditional GWAS is designed for univariate phenotypes, in which the difference in these phenotypes is easy to recognize and measure, such as disease/normal or high/low. However, in high-dimensional data, differences exist, but it is extremely difficult to explicitly describe or quantify. For example, using real life photos, our eyes can easily distinguish different objects in the photos, such as cats or dogs, but it is almost impossible to quantitatively separate them using intensity values of the images. This is a fundamental property of high-dimensional data. Therefore, we believe it is necessary to expand conventional GWAS so that it can take on high-dimensional data such as images. Here the key idea is to cast the association problem under a classification framework. If exposure to genetic mutation led to MR images with a difference that is recognizable by a trained classifier, then we believe it is likely that there is a strong association between this variant and brain-related phenotypes. Although we do not know immediately what exact phenotype is being impacted by the variant, we are nevertheless confident that the impacts of these variants on the MR images are substantial enough to be noticeable by artificial intelligence (AI). Therefore, we believe our strategy extends the definition of phenotypes, and has the potential to uncover novel genotype-phenotype associations and is complementary to existing GWASs. Results obtained when applying our strategy on ADNI MR images support our hypothesis.

Our proposed method addresses a significant limitation of traditional GWAS, which are primarily designed for univariate traits. This design limitation renders traditional GWAS inadequate for handling high-dimensional traits, such as MR image data. Unlike traditional GWAS, which employs a hypothesis testing-based strategy, our method utilizes a classification-based strategy, which makes it more general and versatile.

It is important to note that we do not claim our proposed method outperforms traditional GWAS. Instead, we emphasize that our strategy enables the execution of GWAS on high-dimensional traits, such as full-frame images, which traditional GWAS cannot handle. Thus, our method complements traditional GWAS, expanding the scope of genetic association studies to encompass more complex and high-dimensional data types.

The method we proposed is designed for high-dimensional multivariate data whereas traditional GWAS is designed for univariate trait. There are many fundamental differences between univariate and multivariate data. As a result, many of the concepts we take for granted in traditional GWAS need complete rethinking. As an example, heritability is an important concept in genetics that measures genetic influence on phenotypes [59]. Elliott et al. conducted comprehensive analysis of SNP heritability of image-derived phenotypes (IDPs) and found about half of the IDPs show significant SNP heritability [60]. However, it is challenging to directly estimate heritability for high-dimensional traits such as full frame MR images.

Because our proposed method is designed for high-dimensional traits such as full frame MR images, if an associated SNP is identified, then it is likely that this SNP exhibits pleiotropic effects since the network considers multiple areas of the image. Furthermore, these SNPs are likely to affect other traits including neurological or psychiatric disorders due to pleiotropy. Hence follow up studies will likely generate additional insights.

Superficially, our strategy resembles phenome-wide association study (pheWAS), a study design that explores association between SNPs and a wide range of phenotypes [10]. Unlike GWASs, which screen all variants in the genome to identify associations with a phenotype, a PheWAS study focuses on a single variant, and explore its association with a large collection of phenotypes, often clinical diagnoses collected from electronic health record (EHR) [11–15]. The key difference between our strategy and PheWAS is that our method works with high-dimensional phenotypes, such as full-frame MR images as a whole. In contrast, a PheWAS analyzes multiple phenotypes one by one.

Admittedly, our method has room for improvement. First, the size of the dataset used in our study is limited. The data size is not large enough to develop deeper models to enhance its discriminant power. Our future work will involve collecting more data from different sources such as the UK Biobank to improve the performance of the models.

Overfitting is a problem in machine learning, especially when training data is insufficient, models are too complex, or training for too many epochs. For our application, we only used a relatively simple CNN model with limited parameters and the limited number of training epochs, to minimize the chance of overfitting. Furthermore, we only need to compare the relative performance among all the SNPs.

There are four commonly used genetic models for association studies: additive, dominant, recessive and multiplicative [61]. In our study, we assumed a dominant model when we attempted to apply binary classification on MR images. In association studies, a dominant model assumes that having the mutant allele “a” increases the risk [61]. Hence the genotypes “Aa” and “aa” can be pooled together since they are assumed to have the same risk which is different from the “AA” genotype. We adopted this model to label the MR images according to these two genotype groups. The reason we chose the dominant model is because for many SNPs, the mutant allele is rare, hence the “Aa” and “aa” genotypes have low frequency. By combining them, we reduce the impact of class imbalance which is known to cause problems when the training data is limited. For the additive model, which is the default choice in traditional, testing-based GWAS, all three genotypes are assumed to have different effect on the phenotype. This will lead to a three-class classification problem. Because for SNPs with low or moderate MAF, the number of samples that belong to groups corresponding to genotypes “Aa” and “aa” are often quite limited, this will adversely affect the performance of the CNN classifier, especially when the size of the overall training set is limited. Therefore, we decided not to pursue this genetic model.

We also tested an alternative genetic model which is the recessive model. In this model, two copies of the mutant allele are required for increased risk. Therefore, we grouped the AA (homozygous wild type) and the Aa (heterozygote) genotypes into one class as we assume they have the same effect on the phenotype, and the aa (homozygous mutant) genotype is left for the other group. A small scale test showed that this model also works, albeit with slightly worse classification results overall.

Our strategy is also not limited to SNPs. For other types of variants such as copy number variation (CNV), our proposed strategy is readily applicable. Additionally, classes can be defined based on gene-based or region-based mutation profiles of rare variants with appropriate thresholding.

Third, in order to increase the size of the training data, we included multiple MRI scans from the same individual, which is admittedly undesirable, and will likely reduce the discovery power for the present study. We believe this is a temporary problem which will disappear when the sample size of the study cohort is sufficiently large.

Another key limitation of our strategy is the heavy computing cost, because about 900,000 CNN models need to be trained. In order to reduce computational burden, in this study, we only consider 2D brain images. Hence not all information from the original 3D image data is utilized. For future work, more efficient CNN models such as vision transformers[62] will be explored to handle full 3D MR images.

Although performance metrics are important, our study focused primarily on establishing the feasibility of our classification-based GWAS framework rather than solely optimizing for classification accuracy in a single SNP. Training individual CNN models for each SNP with complex architectures would have required significant time investment due to the large number of SNPs (over 300,000). For instance, utilizing complex CNN models would have resulted in an estimated training time of 254 days, whereas our chosen simple CNN model reduced this to 44 days. While the difference in training time for a single task may seem small, it becomes significant when considering the cumulative time required for all SNPs. Furthermore, our experimentation revealed that the simple CNN model achieved comparable performance to more complex models (Table B in S1 Table), and the results were statistically significant. Therefore, we made the decision to utilize the simple CNN model for all classification tasks to streamline the process and expedite the acquisition of results.

Interpretability is indeed a major challenge for our new strategy. To improve the interpretability of the CNN model, we employed the saliency map technique to highlight the brain regions that are associated with certain SNPs. Additionally, our results present novel hypotheses at the SNP level, perhaps functional experiments on these SNPs can be followed up in future studies to shed new insights on these potentially novel genotype-phenotype associations.

To validate our findings, we conducted a validation study by splitting the ADNI dataset into two separate datasets: ADNI 1 and ADNI 2. As of now, there are only a few datasets that contain both genetic data and brain MRI image data. For future work, in order to assess the generalizability of our findings, we plan to test our strategy on independent real datasets.

On the technical side, we acknowledge that our dataset size of 13,722 MRI images may not be sufficient to train a state-of-the-art (SOTA) CNN model from scratch. While it would have been ideal to use a pretrained model from a larger dataset, it is important to clarify that the primary goal of our study was not to develop the best-performing imaging classification model. Instead, our focus was on establishing the feasibility of our classification-based GWAS framework.

To align with this objective, we intentionally opted for a simplified CNN model to streamline our proof-of-principle study. Our aim was to demonstrate that even with a straightforward model architecture, significant classification results could still be achieved. This rationale guided our choice to use the sex classification task as a benchmark and conduct permutation tests to validate the performance of our simplified CNN model.

To the best of our knowledge, this study is the first to propose the classification-based GWAS framework and apply it to MR full frame image data. We believe that our most important contribution is demonstrating that this strategy works. However, there are still many follow-up questions to optimize our method and improve its performance, which we commit to pursuing in our future research efforts.

And finally, our strategy involves classifying close to a million different classification tasks. The ultimate goal is not to improve classification accuracy in individual tasks, but to identify which task is “classifiable”. Analogous to the multiple testing problem, we are facing a “multiple classification” problem. How to make that distinction is an interesting new problem that requires more research effort which we plan to pursue in future studies.

Despite all the challenges, we believe it is beneficial to conduct classification-based GWAS when phenotype data is high-dimensional. By taking into account of the phenotype data in its entirety, our method is able to capture global, higher-order nonlinear associations between variants and brain phenotypes which is complementary to the traditional GWAS. As a result, our method has the potential to identify new association signals and functional SNPs, therefore providing more comprehensive understanding of genotype-phenotype association. Additionally, there is no need to perform complicated feature engineering to get iQTs or IDPs before running GWAS, which requires in-depth knowledge of brain anatomy and mastering of MR imaging technology.

The new classification-based GWAS framework we introduced is rather general and can accommodate a wide variety of high-dimensional, structured phenotypes. There are many diverse complex phenotypes in the real world including other imaging modalities like positron emission tomography (PET), speech signals, and digital signals produced by medical devices such as ultrasounds, PETs, and electroencephalogram (EEG). Such complex, high-dimensional phenotypes have yet to be fully analyzed in their original form. Our method can apply to all of these data. We believe the novel classification-based association study framework can be applied to a full spectrum of high-dimensional phenotypes and will have a broad impact making novel discoveries between genotypes and high-dimensional phenotypes.

## Supporting information

S1 Table Table A. The full list of the simulation study results. The results were obtained by running the simulation study under different values of the mean, sample sizes. Table B. Summary of the basic information of all the subjects that were included in the analysis. Table C. Training time and performance of different models in the sex classification task. Three different CNN architectures were used: LeNet, ResNet50, and Xception. The performance was measured by the macro F1 and MCC of running these models on the test set. Table D. The full list of the classification results of all SNPs in different three orientations. Table E. The full list of the permutation test results for top 500 SNPs in the axial direction and the genomic context annotation of these SNPs. Table F. The full list of the permutation test results for top 500 SNPs in the coronal direction and the genomic context annotation of these SNPs. Table G. The full list of the permutation test results for top 500 SNPs in the sagittal direction and the genomic context annotation of these SNPs. Table H. Phenotype-Wide Association Study (PheWAS) annotation of the top 500 SNPs in each of the three directions: axial, coronal and sagittal. Table I. The overlap of the three lists of top SNPs ranked by MCC. Table J. The overlap of the three lists of top SNPs ranked by macro F1 score.
(XLSX)

S1 Fig Overview of the ADNI subjects included in this study. **(A)** Histogram displaying the distribution of the number of MRI scans per subject. **(B)** Histogram showing the number of subjects per age group. **(C)** Pie chart representing the proportion of diagnosis in the ADNI cohort, including Alzheimer’s disease (AD), cognitively normal (CN), mild cognitive impairment (MCI), early mild cognitive impairment (EMCI), late mild cognitive impairment (LMCI), and subjective memory complaint (SMC). **(D)** Pie chart illustrating the proportion of male and female individuals in the ADNI cohort.
(PDF)

S2 Fig Scatter plot showing the relationship between the classification performance and the two sample t-test p-values under different simulation settings. The x-axis is -log10 scale of the t-test p-values. The y-axis is the classification performance (MCC and macro F1). N = 3000, 4000, 5000, 6000, 7000. The dashed line is the GWAS threshold of 5e-8. **(A)** Dots are colored by the difference between the mean of the two groups. **(B)** Dots are colored by dfference in the proportion of the majority class.
(PDF)

S3 Fig Scatter plot showing the relationship between the Kullback-Leibler divergence (KLD) and Matthew’s correlation coefficient (MCC) (lower part), the two-sided t-test p-values (upper part) under different simulation settings. The y-axis of the upper part is -log10 scale of the t-test p-values. Simulations are performed under different major class proportions: 0.5, 0.6, 0.7 and 0.8.
(PDF)

S4 Fig Genomic annotation of the top 500 SNPs from classification-based GWAS conducted on three different planes—axial, coronal, and sagittal. Overall, 1301 unique SNPs were annotated. **(A)** Venn diagram displaying overlap among the three lists of top 500 SNPs ranked by macro F1 score. **(B)** Venn diagram illustrating the overlap among the three lists of genes close to the top 500 SNPs. **(C)** Pie charts showing the proportion of variant types among all the top SNPs annotated. **(D)** Histogram of macro F1 values for all SNPs in the axial, coronal, and sagittal planes.
(PDF)

S5 Fig GTEx tissue enrichment of genes nearby (within 10kb upstream and downstream to) the top 500 SNPs in three different planes. **(A)** Axia, **(B)** Coronal, **(C)** Sagittal. The top 20 tissue-specific enrichments are shown for each plane. The enrichment was calculated based on the GTEx data with online tool: https://maayanlab.cloud/Enrichr/.
(PDF)
