# Supplementary material for: A novel classification framework for genome-wide association study of whole brain MRI images using deep learning
Source: PLoS Comput Biol. 2024 Oct 15;20(10):e1012527. doi: 10.1371/journal.pcbi.1012527 (PMC11508069; doi:10.1371/journal.pcbi.1012527)
Supplement: S1 Fig — (A) Histogram displaying the distribution of the number of MRI scans per subject. (B) Histogram showing the number of subjects per age group. (C) Pie chart representing the proportion of diagnosis in the ADNI cohort, including Alzheimer’s disease (AD), cognitively normal (CN), mild cognitive impairment (MCI), early mild cognitive impairment (EMCI), late mild cognitive impairment (LMCI), and subjective memory complaint (SMC). (D) Pie chart illustrating the proportion of male and female individuals in the ADNI cohort. (PDF) [file pcbi.1012527.s002.pdf]

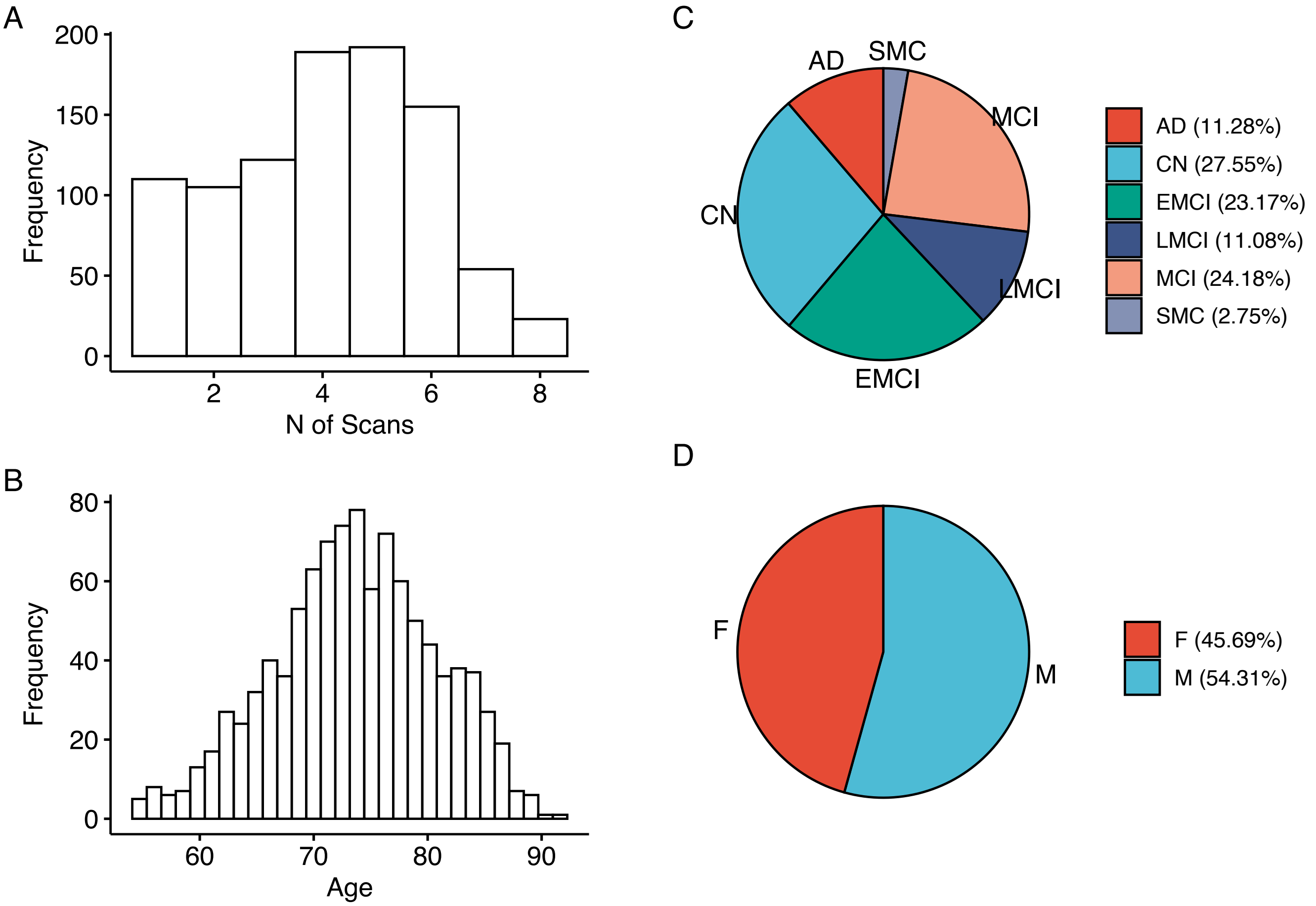

S1 Fig. Overview of the ADNI subjects included in this study. (A) Histogram displaying the distribution of the number of MRI scans per subject. **(B)** Histogram showing the number of subjects per age group. **(C)** Pie chart representing the proportion of diagnosis in the ADNI cohort, including Alzheimer's disease (AD), cognitively normal (CN), mild cognitive impairment (MCI), early mild cognitive impairment (EMCI), late mild cognitive impairment (LMCI), and subjective memory complaint (SMC). **(D)** Pie chart illustrating the proportion of male and female individuals in the ADNI cohort.
