# Supplementary material for: A novel classification framework for genome-wide association study of whole brain MRI images using deep learning
Source: PLoS Comput Biol. 2024 Oct 15;20(10):e1012527. doi: 10.1371/journal.pcbi.1012527 (PMC11508069; doi:10.1371/journal.pcbi.1012527)
Supplement: S2 Fig — The x-axis is -log10 scale of the t-test p-values. The y-axis is the classification performance (MCC and macro F1). N = 3000, 4000, 5000, 6000, 7000. The dashed line is the GWAS threshold of 5e-8. (A) Dots are colored by the difference between the mean of the two groups. (B) Dots are colored by dfference in the proportion of the majority class. (PDF) [file pcbi.1012527.s003.pdf]

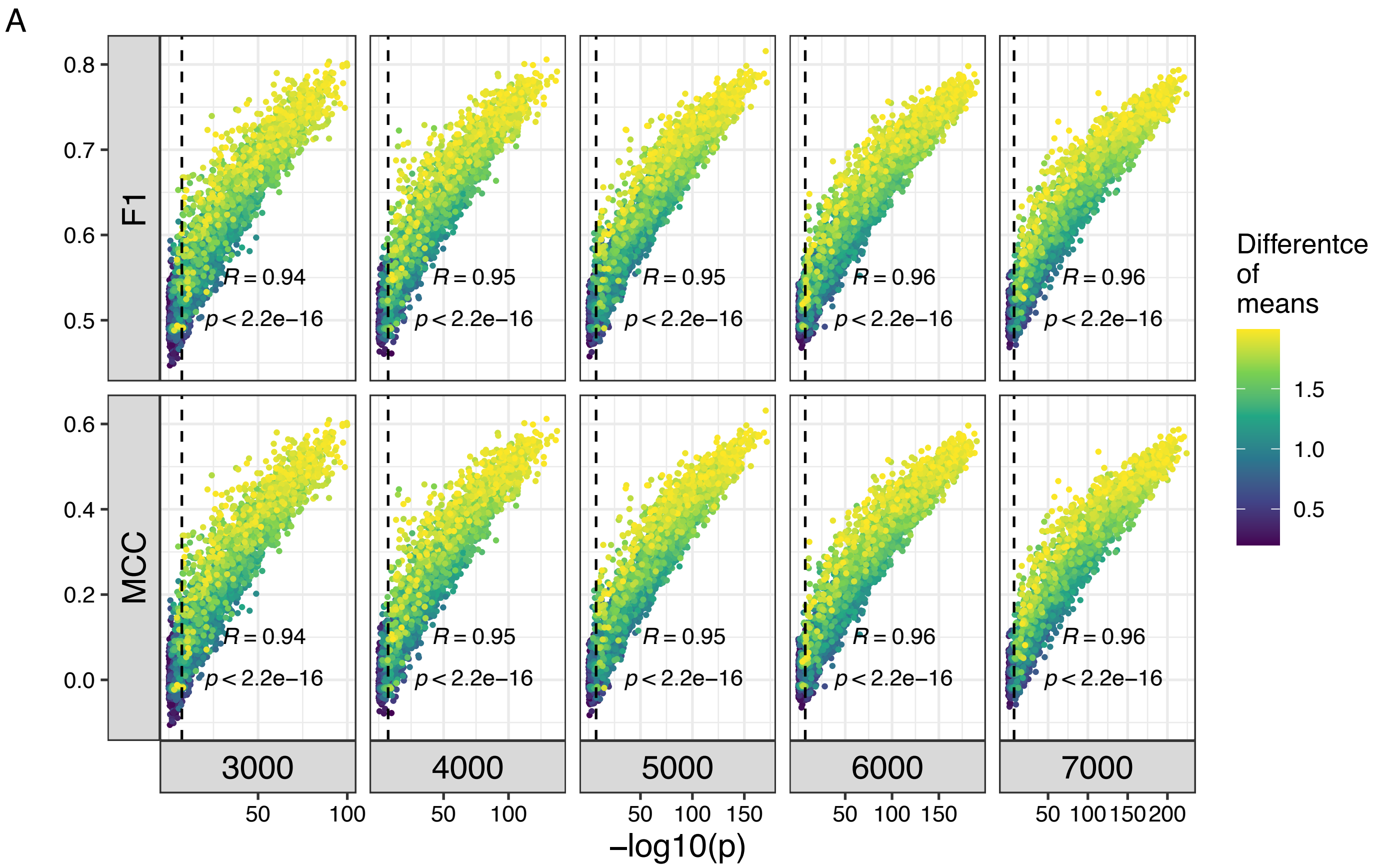

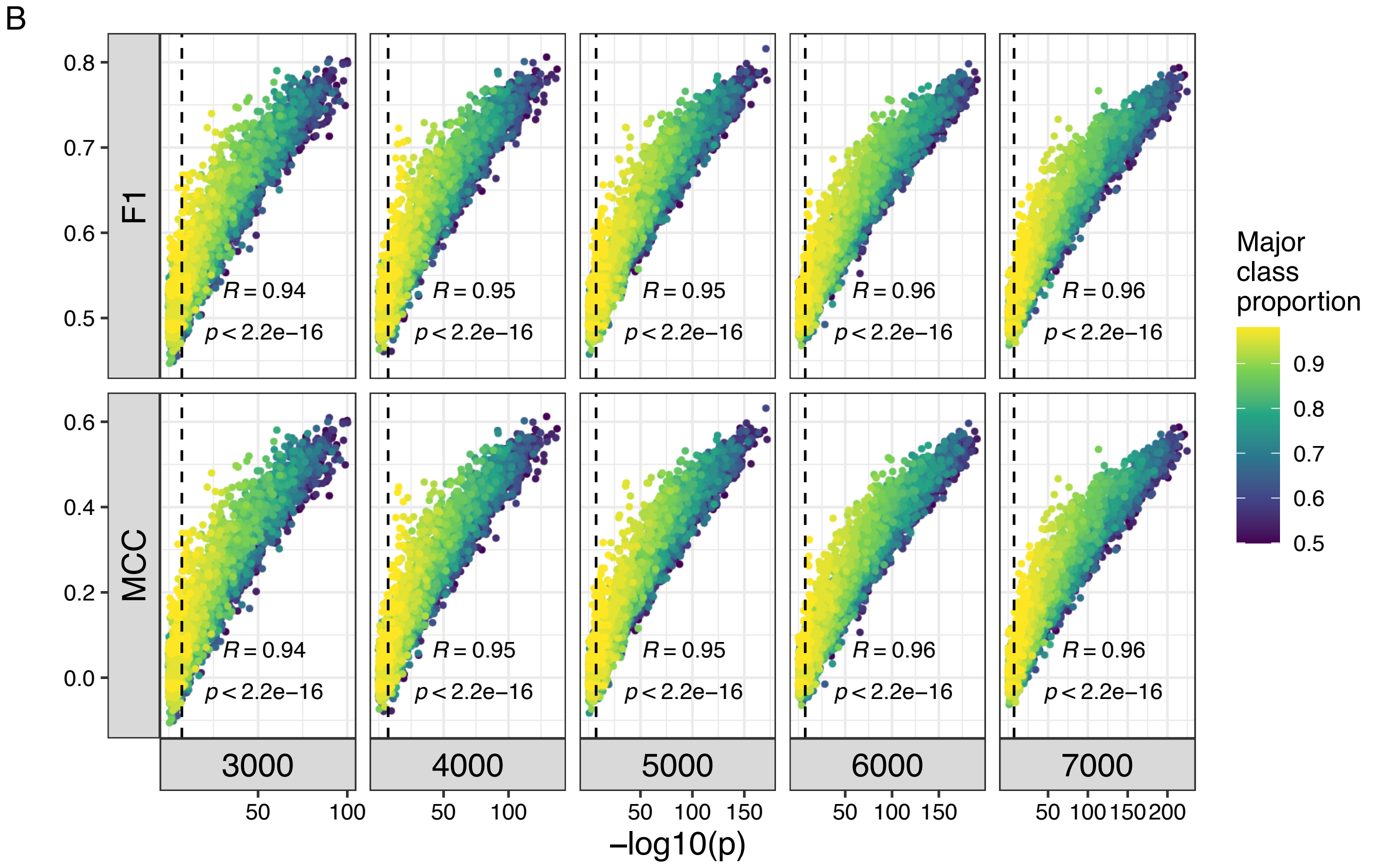

S2 Fig. Scatter plot showing the relationship between the classification performance and the two sample t-test p-values under different simulation settings. The x-axis is - log10 scale of the t-test p-values. The y-axis is the classification performance (MCC and macro F1). N = 3000, 4000, 5000, 6000, 7000. The dashed line is the GWAS threshold of 5e-8. **(A)** Dots are colored by the difference between the mean of the two groups. **(B)** Dots are colored by dfference in the proportion of the majority class.
