# Supplementary material for: A novel classification framework for genome-wide association study of whole brain MRI images using deep learning
Source: PLoS Comput Biol. 2024 Oct 15;20(10):e1012527. doi: 10.1371/journal.pcbi.1012527 (PMC11508069; doi:10.1371/journal.pcbi.1012527)
Supplement: S3 Fig — The y-axis of the upper part is -log10 scale of the t-test p-values. Simulations are performed under different major class proportions: 0.5, 0.6, 0.7 and 0.8. (PDF) [file pcbi.1012527.s004.pdf]

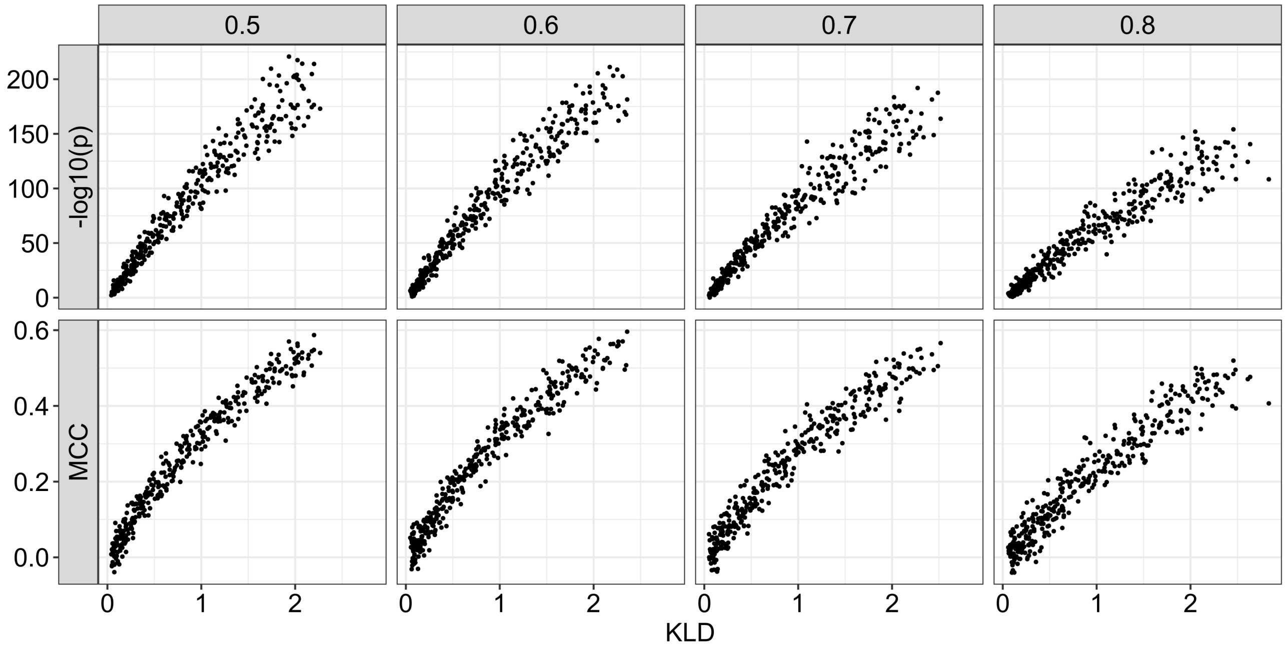

S3 Fig. Scatter plot showing the relationship between the Kullback-Leibler Divergence (KLD) and MCC (lower part), the two-sided t-test p-values (upper part) under different simulation settings. The y-axis of the upper part is -log10 scale of the t-test p-values. Simulations are performed under different major class proportions: 0.5, 0.6, 0.7 and 0.8.
