# Supplementary material for: A novel classification framework for genome-wide association study of whole brain MRI images using deep learning
Source: PLoS Comput Biol. 2024 Oct 15;20(10):e1012527. doi: 10.1371/journal.pcbi.1012527 (PMC11508069; doi:10.1371/journal.pcbi.1012527)
Supplement: S4 Fig — Overall, 1301 unique SNPs were annotated. (A) Venn diagram displaying overlap among the three lists of top 500 SNPs ranked by macro F1 score. (B) Venn diagram illustrating the overlap among the three lists of genes close to the top 500 SNPs. (C) Pie charts showing the proportion of variant types among all the top SNPs annotated. (D) Histogram of macro F1 values for all SNPs in the axial, coronal, and sagittal planes. (PDF) [file pcbi.1012527.s005.pdf]

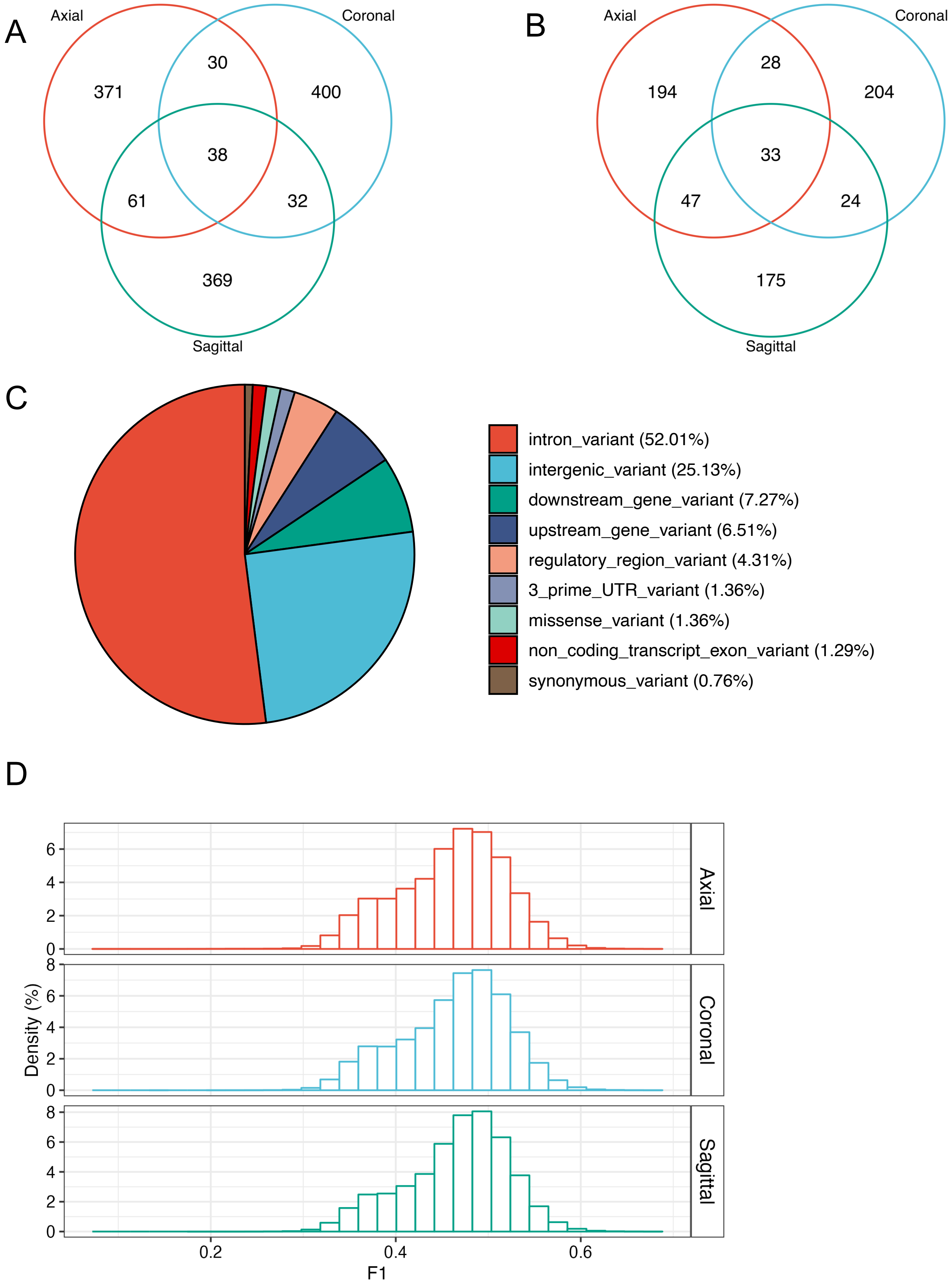

S4 Fig. Genomic annotation of the top 500 SNPs from classification-based GWAS conducted on three different planes--axial, coronal, and sagittal. Overall, 1301 unique SNPs were annotated. **(A)** Venn diagram displaying overlap among the three lists of top 500 SNPs ranked by macro F1 score. **(B)** Venn diagram illustrating the overlap among the three lists of genes close to the top 500 SNPs. **(C)** Pie charts showing the proportion of variant types among all the top SNPs annotated. **(D)** Histogram of macro F1 values for all SNPs in the axial, coronal, and sagittal planes.
