# Supplementary material for: A novel classification framework for genome-wide association study of whole brain MRI images using deep learning
Source: PLoS Comput Biol. 2024 Oct 15;20(10):e1012527. doi: 10.1371/journal.pcbi.1012527 (PMC11508069; doi:10.1371/journal.pcbi.1012527)
Supplement: S5 Fig — (A) Axia, (B) Coronal, (C) Sagittal. The top 20 tissue-specific enrichments are shown for each plane. The enrichment was calculated based on the GTEx data with online tool: https://maayanlab.cloud/Enrichr/. (PDF) [file pcbi.1012527.s006.pdf]

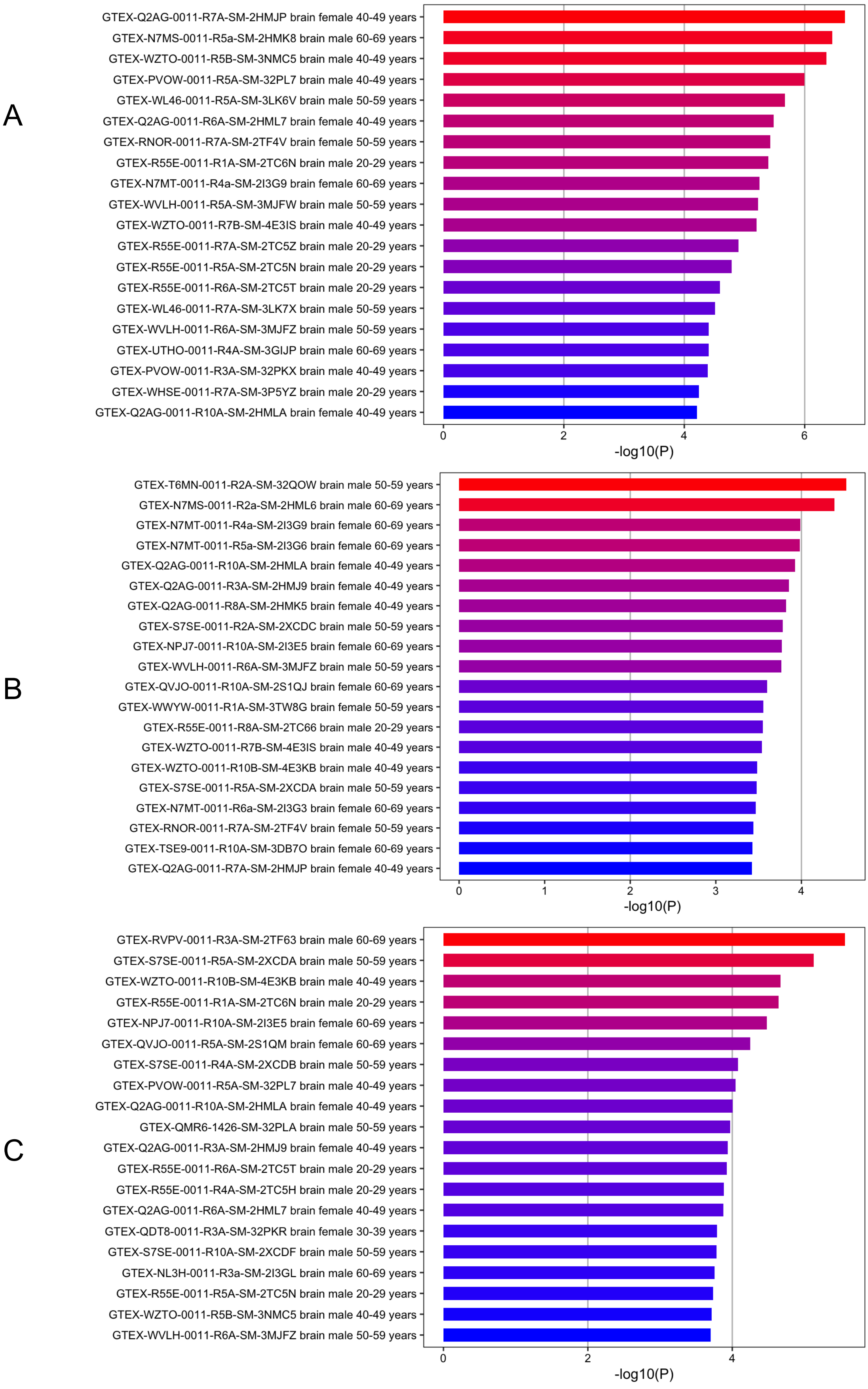

S5 Fig. GTEx tissue enrichment of genes nearby (within 10kb upstream and downstream to) the top 500 SNPs in three different planes. (A) Axia, **(B)** Coronal, **(C)** Sagittal. The top 20 tissue-specific enrichments are shown for each plane. The enrichment was calculated based on the GTEx data with online tool: https://maayanlab.cloud/Enrichr/.
